# Lifetime alcohol use disorder and gambling disorder: Clinical profile and treatment response

**DOI:** 10.1017/S1092852924000300

**Published:** 2024-05-17

**Authors:** Samuel R. Chamberlain, Konstantinos Ioannidis, Jon E. Grant

**Affiliations:** a Department of Psychiatry, Faculty of Medicine, University of Southampton, UK; and NHS Southern Gambling Service / Southern Health NHS Foundation Trust, Southampton, UK; b Department of Psychiatry & Behavioral Neuroscience, University of Chicago, Pritzker School of Medicine, Chicago, IL, USA

**Keywords:** alcohol, gambling, treatment

## Abstract

**Objectives:**

Gambling disorder affects 0.5-2.4% of the population and shows strong associations with lifetime alcohol use disorder. Very little is known regarding whether lifetime alcohol use disorder can impact the clinical presentation or outcome trajectory of gambling disorder.

**Methods:**

Data were pooled from previous clinical trials conducted in people with gambling disorder none of whom had current alcohol use disorder. Demographic and clinical variables were compared between those who did versus did not have lifetime alcohol use disorder.

**Results:**

Of the 621 participants in the clinical trials, 103 (16.6%) had a lifetime history of alcohol use disorder. History of alcohol use disorder was significantly associated with male gender (Relative Risk (RR) = 1.42), greater body weight (Cohen’s D=0.27), family history of alcohol use disorder in first-degree relative(s) (RR = 1.46), occurrence of previous hospitalization due to psychiatric illness (RR = 2.68), and higher gambling-related legal problems (RR = 1.50). History of alcohol use disorder was not significantly associated with other variables that were examined, such as severity of gambling disorder or extent of functional disability. Lifetime alcohol use disorder was not significantly associated with extent of clinical improvement in gambling disorder symptoms during the subsequent clinical trials.

**Conclusions:**

These data highlight that lifetime alcohol use disorder is an important clinical variable to be considered when assessing gambling disorder, since it is associated with several untoward features (especially gambling-related legal problems and prior psychiatric hospitalization). The study design enabled these associations to be disambiguated from current or recent alcohol use disorder.

## Introduction

Gambling disorder affects 0.4-2.4% of the population across the world and is linked to a variety of untoward outcomes such as high levels of comorbidities, disability, bankruptcy, and suicidality.^1^ Gambling Disorder is the first ‘behavioral addiction’ to be recognized in the Diagnostic and Statistical Manual Version 5 (DSM-5),^2^ wherein it is listed in the category of ‘Substance-Related and Addictive Disorders’. This classification of gambling disorder alongside alcohol (and other substance) use disorders was driven by recognition of high etiological, phenomenological, and comorbid overlap across these conditions.^3^ As certain psychoactive substances such as alcohol can lead to addiction in some individuals, so too can certain behaviors – especially gambling.

A recent meta-analysis has indicated that frequency of alcohol use is a potential predisposing factor for gambling disorder, with small-medium effect sizes.^4^ At the same time, however, complex bi-directional relationships are likely to exist. For example, gambling disorder leads to distress and functional impairment, which in turn may lead an individual to develop problematic alcohol use or an exacerbation of existing use. One major challenge in the alcohol use disorder literature per se is to differentiate any effects (or associations) due to current (or recent) alcohol use, from those attributable to a history of alcohol use disorder in itself.

Clinicians often suspect that a history of alcohol use disorder may be relevant to the presentation of gambling disorder and its response to treatment. For example, does the person’s lifetime history of alcohol use disorder indicate they are more prone to other addictions – or switching across them? Does the occurrence of lifetime alcohol use disorder mean that subsequent gambling disorder is harder to treat or presents differently (e.g. with greater symptom severity)?

To address previously unanswered questions regarding lifetime alcohol use disorder and its relationship to gambling disorder, we aggregated data from previous clinical trials for gambling disorder, which excluded individuals with recent/current alcohol use disorder. Associations between lifetime (but not current) alcohol use disorder and other variables were characterized. Previous studies have found that gambling disordered adults with current alcohol problems report greater gambling severity^5–6^ and are at an increased likelihood of relapse to gambling problems after treatment^7^. Similarly, a previous study with 150 adults with gambling disorder found that those with a lifetime alcohol use disorder had worse lifetime gambling symptoms, exhibited more impairment in self-control, and greater resistance to externally-motivated treatment approaches than gamblers without a lifetime alcohol use disorder history.^8^ These studies, however, only examined lifetime alcohol problems that were also current and did not examine whether lifetime (but not current) alcohol use disorders had similar associations. Building upon this limited research, we hypothesized that lifetime alcohol use disorder would be associated with worse current gambling symptoms, family history of alcohol use disorder, male gender, and lesser clinical improvement in gambling from clinical trial participation. The findings from this study may provide needed research information as to the longer-term effects of alcohol use disorder on current gambling behavior and suggest important clinical information as to why some adults with gambling disorder are less successful in gambling treatment.

## Methods

### Participants

Data were aggregated from participants who attended clinical trials of pharmacotherapy or psychotherapy for gambling disorder at the University of Chicago and the University of Minnesota, USA. All diagnoses of gambling disorder were made by an experienced board-certified psychiatrist, using the criteria set forth by the Diagnostic and Statistical Manual Version IV (DSM-IV)^9^ and the diagnoses were later confirmed to be consistent with the current requirements for gambling disorder using the DSM-5 criteria.^2^ Diagnosis was made using a validated instrument (see later).

The exclusionary criteria for these studies were: alcohol use disorder in the preceding 3 months (to eliminate the acute effects of alcohol use on symptom presentation and treatment response and allow for the examination of the more chronic effects of lifetime alcohol use disorder); history of psychotic or bipolar disorder (to ensure participant safety during a trial of medications that might destabilize psychotic or bipolar symptoms), any current (past 3 months) illicit drug use (to avoid the confounding effects of other drug use on symptom presentation and treatment outcome), or inability to provide informed consent. Data from multiple previously published clinical trials were included.^10–16^ Trials were from 8 weeks to 16 weeks in duration.

All study procedures were carried out in accordance with the Declaration of Helsinki. The Institutional Review Boards of the University of Minnesota and of the University of Chicago approved the procedures and the accompanying consent forms. After all procedures were explained, all participants provided informed written consent.

### Assessments

A semi-structured rater-administered questionnaire was used to collect detailed information on demographic and clinical features of gambling (e.g., preferred types of gambling, amount of money lost, occurrence of gambling-related legal problems). Lifetime history of alcohol use disorder was assessed using the Structured Clinical Interview for DSM-IV Axis I Disorders (SCID-I).^17^ All participants included in these trials were drawn from settings where multiple types of gambling (i.e., both strategic and non-strategic) are available. To determine preferred form of gambling, participants were asked as part of the semi-structured clinical interview, which form of gambling they preferred. Strategic gambling was defined as games (e.g., cards, sports, and dog/horse-race wagering) in which skill or knowledge may have some impact on outcomes.^18^ Other games such as slots, lottery, and pull tabs, require no skill, and consequently, these are categorized as ‘non-strategic’ gambling.

We undertook the family history method where the proband is asked about psychiatric and substance use problems in their first-degree relatives, despite its methodological limitations,^19^ as this method aligns most closely with how family history is evaluated clinically. When a participant was unsure of a diagnosis, it was not included.

In addition, participants completed the following instruments:

- Structured Clinical Interview for Gambling Disorder (SCI-GD) for diagnosis of gambling disorder.^20^
- Structured Clinical Interview for DSM-IV Axis I Disorders (SCID-I) to identify mainstream psychiatric comorbidities.^17^
- Yale-Brown Obsessive-Compulsive Scale modified for Pathological Gambling (PG-YBOCS), a clinician-administered scale, to quantify symptom severity over the past seven days.^21^
- Gambling Symptom Assessment Scale (GSAS), a self-report scale, to measure overall symptom severity for the past week.^22^
- Hamilton Depression Rating Scale (HAM-D) to measure severity of depressive symptoms.^23^
- Hamilton Anxiety Rating Scale (HAM-A) to measure severity of anxiety symptoms.^24^
- Sheehan Disability Scale (SDS) to measure overall disability / functioning.^25^

### Data Analysis

The baseline demographic and clinical features of those who did and those who did not have lifetime alcohol use disorder were compared using analysis of variance (ANOVA) for continuous variables or likelihood ratio chi-squares for categorical variables. To assist in the interpretation of significant results, we also reported Cohen’s D (for continuous variables) or Relative Risk (RR, for categorical variables). This being an exploratory study where we wished to avoid risk of falsely assuming a variable was not important when it was (i.e. to minimize likelihood of false negatives), statistical significance was defined as p<0.05 (we did however recognize the potential limitations this might pose, especially in terms of risk for type I errors in exploratory studies).

## Results

Of the 621 participants in the clinical trials, 103 (16.6%) had lifetime but not current alcohol use disorder. The demographic characteristics of the two groups are shown in Table 1. Consistent with prior research (Petry et al., 2005), previous history of alcohol use disorder was significantly associated with male gender (RR=1.42), and greater body weight (Cohen’s D=0.27), but not with the other demographic variables that were examined.

Table 2 shows the baseline clinical characteristics relating to gambling in each of the two groups. Lifetime alcohol use disorder was significantly associated with higher occurrence of gambling-related legal problems (RR=1.50), but not with the other gambling-related measures that were considered. In particular, and contrary to previous research,^5–6^ lifetime alcohol use disorder was not associated with worse gambling symptom severity.

The clinical features of the two groups for the other variables are shown in Table 3. Lifetime alcohol use disorder was significantly associated with previous psychiatric hospitalization (RR=2.68) and with a family history of alcohol use disorder in at least one first-degree relative (RR=1.46). It was not associated with the other clinical variables that were considered.

Participants with a lifetime history of alcohol use disorder did not differ significantly from those without such a history in terms of subsequent clinical improvement observed during the clinical trials, either using a self-report (GSAS) (F=2.487, p=0.115) or a clinician-rated outcome measure (PG-YBOCS) (F=0.107, p=0.743), a finding which differs from previous suggestions that outcomes are worse for people with current alcohol use disorder.^7–8^

## Discussion

This study explored baseline and prospective clinical outcome variables associated with lifetime alcohol use disorder in a relatively large sample (N=621) of people with gambling disorder who participated in clinical trials. The nature of the trials meant that current or recent (past 3-month) alcohol use disorder was exclusionary, enabling us to measure associations with lifetime alcohol use disorder without confounding influences related to current/recent alcohol problems.

On one level it is perhaps surprising that the overall rate of lifetime alcohol use disorder was 16.6% in the current sample and not higher. The observed rate is lower than the US National Epidemiologic Survey on Alcohol and Related Conditions (NESARC), which reported lifetime and past-year prevalence rates of alcohol use disorder of 30.3% and 8.5% in the general population.^26^ This could represent differences in the measures used to quantify lifetime alcohol use disorder in the different samples. Another possibility is that the current data analysis excluded people with current/recent alcohol use disorder, since this was part of the requirements to take part in one of the composite clinical trials, and so one would expect lower rates of lifetime use disorder than may otherwise be found in gambling disorder in general. Thus, the relevance of this work is not so much the absolute rate of lifetime alcohol use disorder (as contrasted to population surveys), but rather the associations between lifetime alcohol use disorder and other variables in people with gambling disorder.

It was found that lifetime alcohol use disorder was linked to significantly higher gambling-related legal problems as well as male gender, previous psychiatric hospitalization, and higher weight (average weight of 202 pounds in history of alcohol use disorder versus 190.1 pounds in those without such history). Overall, these findings may suggest a profile of ‘impulsivity’ in explaining the link between lifetime alcohol use disorder and current gambling disorder. Prior meta-analysis found that impulsivity was a predisposing factor for gambling disorder.^4^ A tendency towards unduly hasty or reward-seeking acts may predispose to both gambling disorder and lifetime alcohol use disorder. Although the trials occurred partly prior to the rise of the online gambling phenomenon, impulsivity can also be prominent in young men who gamble online,^27^ which may render this specific gambling phenotype (highly impulsive men with lifetime alcohol use disorder) even more relevant in current times. Another complementary perspective is that our sample of people who had lifetime alcohol use disorder may fit (to a larger degree as compared to the “control” sample) with the ‘type 3’ gambling subtype, per Blaszczynski’s model, which tends to be linked to greater male gender, impulsivity, and greater tendency towards gambling to alleviate stress (and/or to provide meaning in life).^28^

The link between lifetime alcohol use disorder and family history of alcohol use disorder in first-degree relatives is perhaps to be expected. Not only would family history contribute genetic vulnerability towards alcohol use disorder but also it is well-established that environment and family norms are important determinants of addiction propensity. This finding highlights the clinical importance of considering first-degree family history not only of gambling but also other related conditions in patient evaluations.

Contrary to our expectation, across the clinical trials, there was no evidence that people with a lifetime history of alcohol use disorder differed from those without such a history, in terms of symptom improvement associated with clinical trial participation. From the bench to bedside perspective, these findings suggest that, overall, lifetime alcohol use disorder may not impede treatment response in the absence of current/recent alcohol use disorder. This is also in line with recent work in online gambling treatment discontinuation predictors.^29^ Of course, it may be that future treatments could be tailored to better help those with a lifetime of alcohol use disorder taking into account their different clinical profiles. Thus, the broader implications for treatment approaches might be to focus on the underlying “impulsivity” (the specific outlines of how to define and operationalize that may require further research) in terms of both psychological as well as pharmacological treatments. This could mean seeing lifetime alcohol use disorder as a vulnerability marker for a range of behavioral issues and focus treatment on the cognitive domain of impulsivity as a more targeted and cost-effective approach.

While this is one of the first studies to explore variables associated with lifetime alcohol use disorder in clinical trials for gambling disorder, several limitations should be considered. Findings may not generalize to other settings, such as people who never seek treatment for gambling disorder. This being an opportunistic analysis of pooled data from previously conducted clinical trials, we were not able to analyze all measures that may be relevant to understanding the link between lifetime alcohol use disorder and current gambling disorder. In future work it would be useful to collect a broader range of variables such as self-report and cognitive measures of impulsivity and compulsivity; antisocial tendencies; and aspects of upbringing and family norms. This type of future research might allow for more specific biological or psychological treatment targets (rather than a potentially vague diagnosis of “lifetime alcohol use disorder) and open new avenues of investigation that could be beneficial. Lastly, this being a cross-sectional dataset, we cannot infer causality or directionality of effect.

## Conclusions

In conclusion, in people with gambling disorder taking part in clinical trials, lifetime history of alcohol use disorder was associated with male gender, greater gambling-related legal problems, higher previous psychiatric hospitalization, higher weight, and family history of alcohol use disorder in first-degree relative(s). Thus, even if patients do not have a current alcohol use problem, it is clinically relevant and important to enquire about lifetime problems since this can influence the clinical profile. At the same time, we did not find any evidence that lifetime alcohol use disorder was linked to worse outcomes from clinical trials, in terms of improvement in gambling symptom severity.

## Figures and Tables

**Table 1:** Demographic characteristics of adults with gambling disorder and lifetime alcohol use disorder.

	Lifetime history of alcohol use disorder?						
	No (N=518)		Yes (N=103)				
	Mean / N	Std Dev / %	Mean / N	Std Dev / %	F	P	
**Age, years**	46.9	11.5	48.6	11.2	1.8977	0.169	
**Sex**					13.551	0.0002	**
Female	278	53.7%	35	34.0%			
Male	240	46.3%	68	66.0%			
**Racial-ethnic group**					2.602	0.7611	
Caucasian	401	85.0%	81	85.0%			
African American	39	8.3%	9	9.4%			
Latino/Hispanic	17	3.6%	2	2.1%			
Asian	7	1.5%	2	2.15			
Native American	5	1.1%	2	2.1%			
Other	3	0.6%	0	0%			
**Education level**	3.1	1.0	3.1	0.9	0.0755	0.7837	
**Height, inches**	67.7	4.0	68.0	3.6	0.1972	0.6572	
**Weight, pounds**	190.1	42.9	202.1	49.0	4.8611	0.0279	*
**Smoker?**					0.931	0.3347	
No	149	48.5%	34	42.5%			
Yes	158	51.5%	46	57.5%			

Statistical tests are analysis of variance except where indicated LR = Likelihood ratio chi-square test. Education level is a score reflecting the highest level of education obtained to date, ranging from 0 (did not complete initial basic schooling) through to 5 (higher degree completed). For relationship data, presented as single vs not for simplicity for analysis carried out for full categories (e.g. single, married, cohabiting, etc.) Note that total cell sizes per group may differ due to missing data for some variables.

* p<0.05.

**Table 2:** Clinical characteristics related to gambling for adults with gambling disorder and lifetime alcohol use disorder.

	Lifetime history of alcohol use disorder?						
	No (N=518)		Yes (N=103)				
	Mean / N	Std Dev / %	Mean / N	Std Dev / %	F	P	
**US Dollars lost to gambling in the past year**	23597	32148	30534	31887	2.6633	0.1036	
**GSAS**	34.8	10.2	35.1	11.4	0.0452	0.8318	
**Age when first started to gamble, years**	26.7	13.0	25.6	14.7	0.5924	0.4418	
**Duration of Untreated Illness, years**	10.2	8.4	10.3	9.3	0.0190	0.8903	
**Gambling-related legal problems**					8.861	0.0029	**
No	175	61.2%	31	41.9%			
Yes	111	38.8%	43	58.1%			
**Previous Gambling Treatment**					0.019	0.8906	
No	258	55.5%	54	56.3%			
Yes	207	44.5%	42	43.8%			
**Family history of gambling disorder (1’ relative)**					1.231	0.2673	
No	159	52.0%	36	45.0%			
Yes	147	48.0%	44	55.0%			

Statistical tests are analysis of variance except where indicted LR = Likelihood ratio chi square test. GSAS = Gambling Symptom Assessment Scale. Note that total cell sizes per group may differ due to missing data for some variables.

* p<0.05,

** p<0.01.

**Table 3:** Psychiatric characteristics in adults with gambling disorder and lifetime alcohol use disorder.

	Lifetime history of alcohol use disorder?						
	No (N=518)		Yes (N=103)				
	Mean / N	Std Dev / %	Mean / N	Std Dev / %	F	p	
**Previous psychiatric hospitalizations**					4.969	0.0258	*
No	245	95.3%	63	87.5%			
Yes	12	4.7%	9	12.5%			
**Number of current comorbidities (mainstream mental disorders)**					4.942	0.2933	
0	324	69.5%	56	59.0%			
1	101	21.7%	30	31.6%			
2	34	7.3%	8	8.4%			
3	6	1.3$	1	1.1%			
4	1	0.2%	0	0.0%			
**HAMA**	7.8	4.6	8.5	4.8	1.0789	0.2997	
**HAMD**	7.3	4.0	8.4	4.5	3.5734	0.0596	
**Sheehan Disability Scale**	16.3	6.7	14.9	6.2	2.2581	0.1337	
**Family history of alcohol use disorder (1’ relative)**					15.965	<0.0001	* *
No	146	47.9%	19	23.8%			
Yes	159	52.1%	61	76.3%			

Statistical tests are analysis of variance except where indicated LR = Likelihood ratio chi square test. HAMA = Hamilton Anxiety Rating Scale; HAMD = Hamilton Depression Rating Scale. Note that total cell sizes per group may differ due to missing data for some variables.

* p<0.05.
